# Is TEVAR an Effective Approach to Prevent Complications after Surgery for Aortic Dissection Type A? A Systematic Review

**DOI:** 10.3390/healthcare12131263

**Published:** 2024-06-25

**Authors:** Nikolaos Schizas, Georgia Nazou, Ilias Samiotis, Constantine N. Antonopoulos, Dimitrios C. Angouras

**Affiliations:** 1 4th Cardiac Surgery Department, Hygeia Hospital, 151 23 Marousi, Greece; 2 Department of Cardiac Surgery, Medical School, National and Kapodistrian University, 157 72 Athens, Greece; dangouras@yahoo.com; 3 Anesthesiology Department, Evangelismos General Hospital, 106 76 Athens, Greece; 4 Cardiovascular and Thoracic Surgery Department, Evangelismos General Hospital, 106 76 Athens, Greece; 5 Department of Vascular Surgery, Medical School, National and Kapodistrian University, 157 72 Athens, Greece; kostas.antonopoulos@gmail.com

**Keywords:** aortic remodeling, type A dissection, residual dissection, TEVAR, false lumen

## Abstract

**Introduction:** A residual false lumen after treatment for Aortic Dissection type A (AD) has been associated with early complications, such as A malperfusion or rupture and mid-term or delayed complications, such as aneurysm formation or dissection expansion. Thoracic Endovascular Aortic Repair (TEVAR) is considered an effective solution by several surgical teams to prevent future complications. In this systematic review, all published data regarding the implementation of TEVAR after previous treatment for AD were collected in order to investigate indications, methods, clinical outcomes and aortic remodeling in these patients. **Methods**: The aim of this study was to investigate the indications, the methods and the efficacy of TEVAR usage after surgical treatment of AD. Data for this study were collected from four widely used medical databases (MEDLINE, SCIENCE DIRECT, GOOGLE SCHOLAR, OVID). All the results for each database were recorded and were analyzed with a systematic method. Techniques and clinical outcomes were investigated. Aortic remodeling was evaluated based on the following parameters in these studies: aortic diameter, true lumen diameter, false lumen diameter, false lumen thrombosis and false lumen patency. **Results:** The results obtained from the search among all databases comprised 1410 articles and of these articles 9 were included in the review. The majority of the studies were retrospective (seven out of nine studies), while no study was randomized. The total number of patients was 157 and 133 of them (84.7% of patients) were treated with TEVAR in zone 3 without extension below the diaphragm intraoperatively. Among 142 patients, the calculated mortality rate was 12.7% (18 of 142 patients), with 2.8% (4 of 142 patients) presenting with stroke. The percentage of patients with total or partial thrombosis combined was 65.9% (62 patients in a population of 92). The reintervention rate was 18.7%. **Conclusions:** TEVAR after AD surgery is an approach usually chosen in clinical practice, but the criteria of its usage are uncertain. This method is safe and enhances aortic remodeling with an acceptable reintervention rate. Definite guidelines in this field should be created in order to delineate whether TEVAR after AD surgery is beneficial as a preventive measure to aorta-related complications and to decide under which criteria this approach should be chosen.

## 1. Introduction

The surgical treatment of aortic dissection type A (AD) has sufficiently evolved during the last decade and the outcomes have improved significantly. The mortality rate decreased from 25% in 1995 to 18% in 2013 according to IRAD (International Registry of Acute Aortic Dissection) data [1]. Although surgery was effective in reducing mortality, aorta-related long-term complications continue to be a major cause of concern for patients surgically treated for AD. More specifically, recent evidence showed that almost half of the patients (46.1%) presented with major adverse events related to AD, while 17.3% of patients required reintervention [2]. Residual false lumen after treatment for AD has been associated with early complications, such as malperfusion or rupture and mid-term or delayed complications, such as aneurysm formation or dissection expansion [3]. Indeed, false lumen patency is a negative predictive factor for future aortic degeneration and reinterventions [3].

Despite the fact that the majority of surgeons still believe that the main goal in AD patients is in-hospital survival, mid-and long-term outcomes are undoubtedly critical. Thus, the modern treatment of AD takes into serious consideration possible future reinterventions [4] and aims to primarily address the long-term consequence of patent distal false lumen. In line with this, Thoracic Endovascular Aortic Repair (TEVAR) is considered an effective solution by several surgical teams to prevent future complications. In this systematic review, all published data regarding the implementation of TEVAR after previous treatment for AD were collected in order to investigate indications, methods, clinical outcomes and aortic remodeling in these patients.

## 2. Materials and Methods

Data for this study were collected from four widely used medical databases (MEDLINE, SCIENCE DIRECT, GOOGLE SCHOLAR, OVID). The key words used were aortic remodeling, type A dissection, residual dissection, TEVAR, and false lumen, and we included articles published in English up to 31 December 2023. All the results for each database were recorded and were analyzed by two researchers in the same method from 1 January 2024 to 15 January 2024. The search algorithm was: ((Aortic remodeling)) AND ((Type A aortic dissection) OR (DeBakey TYPE I aortic dissection)) AND ((Residual dissection) OR (False lumen)) AND ((TEVAR) OR (Endovascular aortic repair)). Firstly, abstracts of the articles were evaluated regarding relevance with the topic. Subsequently, relevant articles were fully reviewed and eligible articles were included in the review.

The inclusion criteria consisted of studies in which TEVAR was used for the treatment of residual false lumen after open surgical treatment for AS. Prospective and retrospective studies were included. In studies with various methods or comparative studies, only the patients receiving TEVAR were studied. The exclusion criteria for this review were: (1) studies in which no implementation of TEVAR was performed, (2) Frozen or Elephant Trunk procedure (3), and no follow-up among patients who had TEVAR after surgical repair for AD. Only data from patients who fulfilled the inclusion criteria for this review were included in the final analysis.

## 3. Results

**Figure 1:**
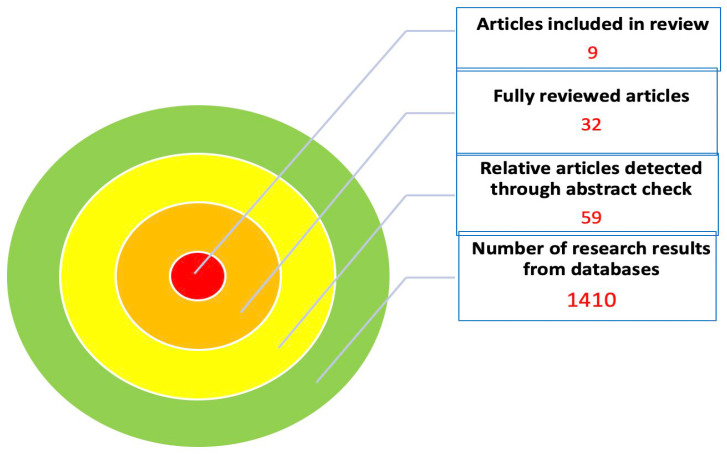
Flow diagram of the article selection process.

A total of 1410 articles were obtained from the search among all databases. A detailed presentation of the number of publications from each database is presented in Table 1. After the initial screening, 59 articles were relevant to the topic of the review. After a review of the abstracts, we detected 32 articles with high relevance, which were fully reviewed. Among these, seven studies were finally identified as eligible for inclusion in the review, while two more studies were found through the author’s secondary search of the references of the included manuscripts (Table 1) [2,5,6,7,8,9,10,11,12]. Additionally, studies with non-randomized and comparative data were evaluated for their quality with the Newcastle—Ottawa Scale [13]. The outcomes of article evaluation are presented in Table 2 according to the aforementioned scale (Table 2). Out of five studies in total, two were categorized as medium-quality studies (four and six stars) and three as high-quality studies (seven stars for each study). Consequently, the number of publications included in the review was nine and the flow chart is depicted in Figure 1. 

All articles were published between 2008 and 2022, including data from 1994 to 2019. The duration of each study varied from 1 year to 10 years. The majority of the studies were retrospective (seven out of nine studies), while no study was randomized (Table 3). The total number of patients was 157 and 133 of them (84.7% of patients) were treated with TEVAR in zone 3 without extension below the diaphragm intraoperatively. The criteria for TEVAR vary a lot and the indications implemented in each study are presented in Table 3. Similarly, the techniques, the devices and the grafts used vary too.

**Figure 2:**
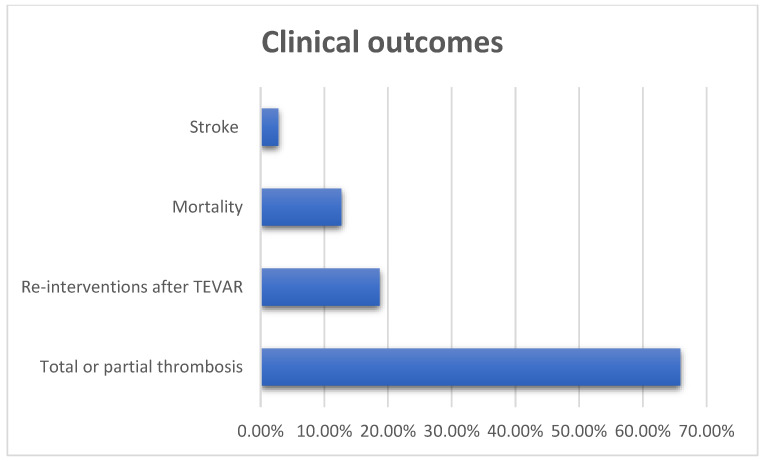
Clinical outcomes after TEVAR for the treatment of residual false lumen.

Studies also vary a lot in design and reporting evidence. As regards pre-TEVAR patients’ characteristics, CT findings and clinical status are poorly reported, and, as a result, this heterogeneity makes a comparison of the patient groups inappropriate. Clinical outcomes after TEVAR are reported in seven of the nine studies. In one study, mixed data with other methods are reported (Jakob et al.) [5] and in another study the clinical outcomes are not reported at all [2] (Table 4). Therefore, among 142 patients, the calculated mortality rate was 12.7% (18 of 142 patients), with 2.8% (4 of 142 patients) presenting stroke (Table 4, Figure 2).

The follow-up examination period ranged from 4.84 months to 8 years, while in almost all studies the aortic remodeling investigation was performed in 12 months after TEVAR. Aortic remodeling was evaluated based on the following parameters in these studies: aortic diameter, true lumen diameter, false lumen diameter, false lumen thrombosis and false lumen patency (Table 5).

Only three studies reported aortic diameter measurements in the follow-up examinations. Pochettino et al. [7] and Sultan et al. [9] reported very satisfying outcomes as regards aortic diameter in all cases, while 4 out of 11 patients presented aortic diameter increase in Di Tommaso et al.’s study [10]. Two studies included information about true lumen diameter, both indicating that TEVAR contributed to the true lumen diameter and area increase. Evidence about false lumen diameter were reported from three studies, showing that TEVAR was associated with a decrease in false lumen diameter. More specifically, Shimamura et al. [6] found the complete disappearance of false lumen in 30.3% and Pochettino et al. [7] in 17% of cases. The patency of the false lumen was reported as a parameter of aortic remodeling in three studies. A total of 22 cases out of 60 (percentage 36.6%) had a patent false lumen. Most researchers had chosen to include the degree of thrombosis in the results of their studies (seven of nine studies mention this information). The percentage of patients with total or partial thrombosis combined was 65.9% (62 patients in a population of 92) (Table 5).

Evidence on reinterventions after TEVAR was also included in the majority of the studies (five studies out of nine). The reintervention rate was 18.7% (14 cases out of 75) (Figure 2). It is worth mentioning that two patients were submitted to open surgery, one patient was treated with left subclavian artery occlusion with coils due to retrograde perfusion and the rest were treated with endovascular repair (Table 5).

## 4. Discussion

The deployment of a stent graft in the descending aorta to improve aortic remodeling and avoid aorta-related complications after surgical treatment for AD has been increased. This strategy is widely used in clinical practice but there are no definite guidelines and criteria for its implementation. A significant heterogeneity among the eligible studies regarding the indication for TEVAR was captured in our review. The indications vary a lot, but several predictors of negative aortic remodeling are commonly acceptable. Among these predictors, an aortic diameter > 35 mm, a false lumen diameter > 22 mm and an entry tear >1 mm were reported by the eligible studies [14]. Undoubtfully, the development of certain criteria for TEVAR usage after aortic dissection may offer the better management of such cases and improved documentation of the outcomes. We believe that the usage of TEVAR may be considered in patients with a total aortic diameter of more than 40 mm, a patent false lumen, the presence of an entry tear in zones 3 and/or 4 (based on Ishimaru zones) and a diameter of a false lumen more than 20 mm, but we have to highlight the fact that there is not enough evidence to support these recommendations at present.

In the majority of patients receiving TEVAR after surgery for AD, TEVAR was placed intraoperatively in approximately 84.7% of the patients, but preoperative evidence is poorly reported in almost all studies. Hence, it is not feasible to detect the profile of a patient receiving TEVAR intraoperatively. Another point that should be underlined is the follow-up after surgically treated AD patients. According to the reported evidence, most researchers did not stick to a certain follow-up protocol. It should be highlighted that close follow-up with repetitive Computed Tomography Angiography (CTA) is suggested in all patients surgically treated for AD, regardless of the extent of the false lumen. More specifically, CTA should be performed in the first month, 6 months and 1 year after surgery, and then annually for at least the first five years from the surgery, except in the cases where the findings suggest an even closer follow-up [15]. CTA is the preferred method of follow-up while Magnetic Resonance Angiography (MRA) is a reliable alternative.

Luminal communication between the true and false lumen is related to aortic diameter increase and consequently to reintervention rate. The 3-year freedom from reintervention is 96% when communication is absent and 47% in the presence of luminal communication [16]. Evidence shows that communication occlusion and the stenting of the descending aorta enhances aortic remodeling, as the partial or complete thrombosis of the false lumen reaches 88.9% [17]. In this review, partial or complete thrombosis was achieved in 65.9% of cases and the patency of false lumen was present in 34.1% (Table 5).

The reintervention rate was 18.7% and 2 out of 14 patients were submitted to open surgery. In a retrospective study including 534 patients surgically treated for AD, 37 of them required reintervention [18]. Of these patients, 30 were submitted to open surgery and 7 were in endovascular repair, while additional interventions were necessary in several patients [17]. These findings suggest that in cases of TEVAR implementation after surgery for AD, endovascular repair is the main approach for reintervention (2/14 cases), while in cases that no intervention is performed after AD surgery, open extended surgeries are required (30/37 cases). Other methods that aim to improve aortic remodeling such as the Frozen Elephant Trunk hybrid method are associated with a high rate of reintervention, both intentional or urgent. More specifically, the percentage of reinterventions reaches 33% while the usage of TEVAR after FET shows satisfactory outcomes regarding technical success and aorta remodeling [19,20]. Although indications and the extent of intervention are different between TEVAR after AD surgery and FET after AD, the findings show that the reintervention rate is lower in cases of TEVAR.

## 5. Limitations

As the studies were heterogenous, statistical analysis was not feasible. The statistical analysis would be biased and may lead to misleading conclusions. This fact is mainly due to (a) differences in the methods among studies, (b) differences in indications for TEVAR, (c) differences in the evidence presentation, (d) great variability in the follow-up period and in the choice of clinical or depicting outcomes presentation and (e) the deficit of randomized multicenter clinical trials on this field. Hence, in order to avoid weak or false conclusions, we present the evidence after meticulous investigation without statistical analysis.

## 6. Conclusions

TEVAR after AD surgery is an approach usually chosen in clinical practice, but the criteria of its usage are uncertain. This method is safe and enhances aortic remodeling with an acceptable reintervention rate. Definite guidelines on this field should be created in order to delineate whether TEVAR after AD surgery is beneficial as a preventive measure to aorta-related complications and to decide under which criteria this approach should be chosen.

## Figures and Tables

**Table 1:** Table with the data of the article selection process.

Databases-Sources of Review	Number of Results	Relative Articles	Fully Reviewed Articles	Articles Included in Review
PUBMED	23	18	5	3
SCIENCE DIRECT	327	17	9	1
GOOGLE SCHOLAR	1010	13	9	2
OVID	50	11	9	1
AUTHOR’S ADDITIONAL	-	-	-	2
Total	1410	59	32	9

**Table 2:** Evaluation of the quality of comparative and non-randomized studies included in the systematic review. Studies between 4 and 6 stars are considered as medium-quality and studies with more than 7 stars as high-quality studies.

Study Name	Selection	Comparability	Outcome	Total Stars
Morishita et al. [11]	**	*	***	6
Jakob et al. [5]	**	*	*	4
Preventza et al. [8]	***	*	***	7
Sultan et al. [9]	***	*	***	7
Li et al. [12]	***	*	***	7

*: Star granted for quality of the study according to Newcastle—Ottawa. Each star is one item.

**Table 3:** All studies regarding TEVAR usage after surgically treated aortic dissection type A. Information regarding the type of study, study period, number of patients, time of intervention, criteria of TEVAR and technical parameters.

Publication	Year	Type of Study	Study Period	Number of Patients	Time of Intervention	Criteria of TEVAR	Technical Parameters
Jakob et al. [5]	2008	Retrospective observational comparative	2001–2007	4	Intraoperative	De Bakey 1 dissection and findings from CTA that were considered eligible from antegrade TEVAR according to surgeons’ aspects	Talent stent graft
Shimamura et al. [6]	2008	Retrospective observational	1994–2004	29	Intraoperative	Presence of entry tear in distal arch or descending thoracic aorta that could not be treated with hemiarch replacement	Custom made from Gianturco stent and WSL graft
Pochettino et al. [7]	2009	Retrospective observational	2005–2008	24	Intraoperative	Presence of entry tear in distal arch or descending thoracic aorta	GORE TAG graft
Preventza et al. [8]	2014	Prospective invasive—Comparative study (surgical approach changed in 2009)	2005–2012	25	Intraoperative	Malperfusion. Aneurysmal dilation of proximal descending aorta. Extend of dissection to the diagram (Criteria are not specific but defined by the surgeon’s aspect)	GORE TAG graft till October 2011 Conformable TAG device after November 2011
Sultan et al. [9]	2017	Retrospective observational comparative	2006–2014	21	Intraoperative	All patients surviving at least 1 year in whom CT Angiography of 1 month and 1 year could be obtained.	GORE TAG graft
Di Tommaso et al. [10]	2018	Retrospective observational	2009–2015	11	4.7 ± 2.3 years	Aortic diameter > 45 mm. Aortic growth > 5 mm/year Impenting rupture	Bare stent 5 patients Djumbodies Dissection System 6 patients Jotec E-XL
Morishita et al. [11]	2019	Prospective non-randomized trial (Observational on new management strategy)	2015–2016	11	122 (50–197) days	(1) Age < 60 years, (2) Patent false lumen. (3) Aortic diameter > 46 mm	Bare metal stent (Zenith dissection endovascular system) 4 cases required left subclavian artery revascularization
Gaudry et al. [2]	2021	Prospective observational	2017–2019	2	Not defined	Aortic diameter > 55 mm Rapid aortic growth (>10 mm/year). Malperfusion syndrome Aortic rupture	Bare stent STABILISE technique
Li et al. [12]	2022	Retrospective observational comparative	2016–2019	30	Intraoperative	True lumen collapse less than 50%	20 Conform TAG, 5 GORE TAG, 2 Navion, 2 Zenith Diss, 1 Zenith TX2

**Table 4:** Data regarding preoperative findings regarding pre-TEVAR false lumen characteristics and clinical status. Additionally, the clinical outcomes (mortality, stroke) after TEVAR are presented.

Publication	Year	Time of TEVAR	Number of Patients	Follow-Up	Time of Remodeling Investigation after TEVAR	Aortic Diameter	True Lumen Diameter	False Lumen Diameter	False Lumen Thrombosis	False Lumen Patency	Reinterventions
Jakob et al. [5]	2008	Intraoperative	4	23 ± 17 months	12 months and then annually	Not certain due to combined group (Change in treatment during study period)	Not certain due to combined group (Change in treatment during study period)	Not certain due to combined group (Change in treatment during study period)	Not certain due to combined group (Change in treatment during study period)	Not certain due to combined group (Change in treatment during study period)	1 reintervention due to endoleak
Shimamura et al. [6]	2008	Intraoperative	29	8 years	Not defined	Not mentioned	Not mentioned	Data combined with chronic dissections 30.3% complete disappearance and 48.4% in thoracic lesion	Not mentioned	Not mentioned	Not mentioned
Pochettino et al. [7]	2009	Intraoperative	24	Not mentioned	Not defined	For 24 patients with favorable remodeling mean diameter was 29.1 ± 4.3 mm and for 6 patients with fully patent false lumen 38.1 ± 5.3 mm		Fully obliteration of false lumen in 5 patients (17%)	Partial thrombosis in 19 patients (79.2%)	Full patency in 6 patients (20%)	8 patients received additional endovascular repair
Preventza et al. [8]	2014	Intraoperative	25	4.84 months	Not defined	Not mentioned	Not mentioned	Not mentioned	Partially thrombosed in 8 patients Total thrombosis in 2 patients	Patent in 10 patients	1 patient submitted to open surgery
Sultan et al. [9]	2017	Intraoperative	21	12 months	1 year	Maximum diameter at level of T6 with diameter 32.8 ± 8.6 mm	Maximum diameter at left subclavian artery level with diameter 29.8 ± 4.3	Not mentioned	Complete thrombosis chest 15 (71.4%) and abdomen 16 (76.2%)	Not mentioned	Not mentioned
Di Tommaso [10]	2018	4.7 ± 2.3 years	11	5.2 ± 1.9 years	Approximately 5 years	Increase in aortic diameter in 3 patients requiring intervention, 1 patient with aortic enlargement treated conservatively	Not mentioned		4 patients total or partial thrombosis	6 patients	1 patient treated with open surgery 2 patients submitted to TEVAR extension (PETTICOAT technique)
Morishita et al. [11]	2019	122 days	11	12 months	12 months	Not mentioned	Increase 203 mm^2^	Average decrease in false of 276 mm^2^	8 patients total thrombosis 3 patients partial thrombosis		Coil occlusion of LSA due to retrograde perfusion of false lumen
Gaudry et al. [2]	2021	Not defined	2	Not defined	Not defined	Not mentioned	Not mentioned	Not mentioned	Total thrombosis	None	None
Li et al. [12]	2022	Intraoperative	30	32 months	Not defined	Not mentioned	Not mentioned	Not mentioned	Not mentioned	Not mentioned	Not mentioned

**Table 5:** Information regarding follow-up, aortic remodeling parameters and reinterventions.

Publication	Year	Number of Patients	Preoperative False Lumen Diameter	Preoperative Patent False Lumen	Organ Malperfusion (Preoperatively)	Mortality	Stroke
Jakob et al. [5]	2008	4	Not mentioned	Not mentioned	Not mentioned	Not certain due to combined group (Change in treatment during study period)	Not certain due to combined group (Change in treatment during study period)
Shimamura et al. [6]	2008	29	Not mentioned	Not mentioned	7 cases malperfusion of lower extremity	2 deaths in-hospital	0
Pochettino et al. [7]	2009	24	Not mentioned	Not mentioned	3/36 transient paraparesis	5 deaths in-hospital deaths	1
Preventza et al. [8]	2014	25	Not mentioned	Not mentioned	19	3	3
Sultan et al. [9]	2017	21	Not mentioned	Not mentioned	0	0	0
Di Tommaso [10]	2018	11	44.1 ± 2.3	All patent	0	1	0
Morishita et al. [11]	2019	11	Not mentioned	Not mentioned	Not mentioned	Not mentioned	Not mentioned
Gaudry et al. [2]	2021	2	55.7 mm	Not mentioned	0	0	0
Li et al. [12]	2022	30	Not mentioned	Not mentioned	0	4 deaths in-hospital and 3 deaths in follow-up period	0

## Data Availability

The datasets used and/or analyzed during the current study are available from the corresponding author on reasonable request.
